# The mismatch between conventional house price modeling and regulated markets: insights from The Netherlands

**DOI:** 10.1007/s10901-016-9529-y

**Published:** 2016-10-04

**Authors:** Qi Tu, Jan de Haan, Peter Boelhouwer

**Affiliations:** 1 OTB-Research for the Built Environment, Delft University of Technology, Delft, The Netherlands; 2 Statistics Netherlands, The Hague, The Netherlands

**Keywords:** Modeling, Intervention, House prices, Mismatch, The Netherlands

## Abstract

House price modeling has been frequently used to investigate the dynamics of housing markets, especially competitive markets; yet less attention has been given to markets that have experienced considerable interventions. The aim of this study is to demonstrate a mismatch between conventional house price models and the case of the Netherlands and to provide reasons of such mismatch. We first describe and classify the conventional house price models into asset-pricing house price model, stock-flow model, multi-period utility model, and repayment model. These models are subsequently applied to the Netherlands, where considerable government interventions took place. As expected, the empirical results are unsatisfactory to explain the Dutch house price development. The degree of mismatch of the repayment model and the multi-period utility model, however, seems to be fairly limited.

## Introduction

An effective way to investigate the dynamics of house prices is house price modeling, most of which has been applied to the markets where limited intervention is taken into account as the competitive market assumption is usually imposed. This leads to a broad literature of house price models based on the asset-pricing perspective. Adapting a model that originated in the financial sector, Campbell and Shiller (1988a, b), for example, developed an asset-pricing model between house prices and rents, which has been studied extensively ever since, notably by Brunnermeier and Julliard (2008) and Mols and Lindenthal (2011).

Few scholars have modeled price dynamics in a regulated environment due to the complexity of the influences induced by different interventions; instead, more attention is given to the impact from certain restricts. One major focus in the literature is the association between tax regime and the cost of owning housing units (see, e.g., Poterba 1984; Díaz and Luengo-Prado 2008; Figari et al. 2012). Another is the impact of the spillover from land regulations and zoning plans on house prices (see, e.g., Peng and Wheaton 1993; Glaeser et al. 2003; Huang and Tang 2012). The discussion of rent control tends to emphasize the welfare distribution and market efficiency (see, e.g., Gyourko and Linneman 1989; Glaeser and Luttmer 1997; Micheli and Schmidt 2014).

The literature has showed limited attention to the house price modeling in the markets with intervention, which is, however, potentially important as using conventional models may lead to problems. Models for competitive markets are likely to fail to explain prices in a highly regulatory environment, given that the economic principles set up for free markets do not fit. This gap can be explained from the impacts of interventions on house prices. If the classic modeling approach is applied directly to such a market, the corresponding result is expected to be unsatisfactory because a different price pattern caused by the non-market interventions. This illuminates the question whether a mismatch exist between house price modeling and regulated environments; if so, how can the mismatch be interpreted in the presence of intervention.

To answer this question, we use the case the Dutch housing market which has experienced considerable interventions to investigate whether such mismatch exists. We also aim at providing the sources of the mismatch from the perspective of intervention and interpreting the differences generated from the modeling results. The Dutch housing market is chosen because it provides a case of various regulatory structures, such as the strong regulation of rental housing and the favorable tax treatment of the owner-occupancy. Though the definition of regulation is lacking^1^, this paper focuses on interventions comprised of tenure, financial affordability, tax regime, and housing supply.

The rest of the paper is structured as follows. First, we classify the standard approaches to house price modeling in terms of their theoretical emphasis and underlying assumptions, which leads to the asset-pricing, stock-flow, multi-period utility, and repayment models. Subsequently, the general features of interventions are described in Sect. 3. The modeling results of the Dutch housing market and discussion are presented in Sect. 4. We conclude with a discussion of the mismatch in Sect. 5.

## Four conventional approaches to house price modeling

In this section, we classify the conventional house price models into four types: the asset-pricing model, the stock-flow model, the multi-period utility model, and the repayment model. These four categories are distinguished because of their economic principles used in each model type, which does not necessarily mean the four categories are incompatible^2^.

### Asset-pricing model

The house price model generated from asset-pricing theory has a similar ground to that of financial market. Investors are motivated to invest in order to yield returns from both dividends and capital gains; likewise, when households purchase a housing unit, they expect to gain benefits from rent flows accrued in the possessing period and capital gains (Leamer 2002).

The asset-pricing model of house prices provides an intuitive method to connect the cost of owning housing units to rents through introducing the concept of user cost, which by definition is comprised of depreciation cost of the housing unit, maintenance fees, mortgage interest payment, and the capital gains from the housing asset (Poterba 1984). The model presents an association among user cost, ${\text{UC}}_{t}$, the house prices, $P_{t}$, and the rents, $R_{\text{t}}$:

1 $$\frac{{R_{t} }}{{P_{t} }} = {\text{UC}}_{t}$$

This equation clarifies the arbitrage opportunity between renting and owning a housing unit. It provides a benchmark for households to decide whether to undertake owner-occupancy by comparing the cost and benefit. If the rent-to-price ratio exceeds the user-cost rate, renting becomes less attractive than ownership. When the rents equal the costs of owning (Eq. (1) holds), a state of equilibrium is reached. This rent-to-price relationship fits well in markets where the rental regulation is absent or limited.

The economic support of this price-to-rent relationship is grounded in the dividend-discount model, which was later extended to take account of the time-variate effect on the dividends (Campbell and Shiller 1988a, b). The extended dynamic dividend-discount model was further elucidated by imposing the long-run stationary rent-to-price ratio, leading to the decomposition of rent-to-price ratio at period t as the sum of households’ expected discounted value of rent increase rate, premium, real risk-free interest rate, and a constant term (Campbell et al. 2009). The decomposition provides an elaborate manner of reflecting the dynamic dividend-discount model to the relationship in Eq. (1).

The difficulty of estimating the presented relationship lies in the measure of user cost. Many scholars have applied this method (see, e.g., Buckley and Ermisch 1982; Himmelberg et al. 2005; Brunnermeier and Julliard 2008; Gallin 2008; Mols and Lindenthal 2011), using the similar measure of user cost as proposed by Poterba (1984).

### Stock-flow house price model

Stock-flow model gives special attention to the dynamics of the housing supply sector, using the demand–supply framework. This means the extra housing units added to the housing market plays a significant role in adjusting the path of house prices to a new equilibrium, which is realized from the supply response to the shifts in housing demand.

The concept of the flux of the housing units is highlighted. Both the construction activities and the demolition speed of the existing housing stock are taken into account to calculate the net flows of the housing stock based on the theory of durable goods and stock accumulations proposed by Clower (1954), which was later implemented in the real estate market by Muth (1957). Many other scholars have ever since studied the adjusted forms of the flux of housing stocks. DiPasquale and Wheaton (1992, 1994, 1996) incorporated the flux concept into a stock-flow model for the housing market with two sub-markets: the property market which determines the demand of space, and the asset market that determines the price of housing units. The ‘flow’ element enters the dynamic setting as follows:

2 $$\Delta S_{t} = C_{t} - \sigma S_{t}$$

where $S_{t}$ is the housing stock at the beginning of period *t*, $\Delta S_{t}$ is the increase in the stock during period *t*, $C_{t}$ is the extra added housing units, and σ is the depletion rate of the existing housing stock.

Equation 2 connects the property market to the asset market through the construction activities. Any positive shocks in the demand from the asset market would temporarily lead to an immediate price increase, subsequently stimulating the construction activities. The additional housing units, in turn, would alleviate the extra demand generated from the asset market gradually until a new equilibrium is reached. The equilibrium state can be derived by setting $\Delta S_{t}$ in Eq. (2) equal to zero, resulting in the connection between the amount of housing stock and that of construction, which are further linked through rents and house prices.

### Multi-period utility model

The multi-period utility model is generated from the optimal consumption profile that emphasizes intertemporal allocation of consumption (Yaari 1964). Households choose the amount of housing service and other consumption to maximize the utility, given the wealth constraint. The house price derived from this model specifically emphasizes households’ preferences.

The generated house price model is dependent of the utility function. One appropriate setting is the Stone–Geary utility form extended from Cobb–Douglas function, which additionally includes a minimum amount of consuming each good. This form can reasonably represent the utility generated from housing consumption as the inherent necessity property of housing is taken account by introducing a minimum amount that has to be satisfied for housing consumption. Olsen (1987) applied such utility presentation to consider a household who accumulates wealth and spends on housing and a composite good to maximize the multi-utility function in the Stone–Geary form. The optimal solution leads to explaining the spending on housing in a linear function by the spending on the minimum amounts of housing service and the composite consumption, and the wealth^3^.

### Repayment model

The repayment models concentrate on the restrictions imposed by financial intermediaries as most households rely on applying for a mortgage loan to purchase a house. The maximum amount that financial intermediaries are willing to lend depends on their lending criteria, which in practice is evaluated by the capability of repaying the mortgage loan. The repayment models thus reflect the agreement reached by both parties: banks’ willingness to lend relates house prices to households’ ‘affordability,’ suggesting a binding effect of housing finance on housing demand.

To highlight the maximum borrowing capacity, Madsen (2012), for example, used a maximum lending ratio which was defined as the amount of loan that a household needs to repay to purchase a housing unit divided by the average income to capture households’ demand, assuming a binding effect of the credit restriction. The result for the aggregate market gives the house prices in derived-demand form expressed by the maximum lending ratio, the income, the housing stock, and the adapted mortgage interest rates.

These four types of conventional models are classified because each of them addresses a particular economic principle. There are a variety of extended studies of the conventional models. For instance, McQuinn and O’Reilly (2008) and Addison-Smyth et al. (2009) used the present value of annuity to determine the borrowing limit in the repayment type of model. In this study, we choose the simple representative to discuss the potential reasons for the differences in results.

## The regulatory environment

Regulatory environments can potentially affect the development of house prices and have been realized in different forms, including restrictions on the supply sector such as zoning plans, and policies to influence demand, like the tax deduction policy. We discuss four distinguishing interventions in the regulatory environment^4^, including tenure choice^5^, affordability, tax incentives, and supply restrictions. These regulatory features are selected because each of them represents one of the roles of households, financial intermediaries, government, and constructors, though they are likely to have an interacting impact on influencing the prices.

The tenure choice mainly falls under the rubric of the rental housing system. It encompasses different ways of subsidizing renting, including direct provision of social housing units by authorized institutions and imposition of rent controls as permitted by law. However, rent controls vary in flexibility due to different regimes. Some countries also offer housing allowances directly to tenants who satisfy certain conditions. For instance, the Netherlands introduced an allowance for the demand side as an effort to improve affordability (Elsinga et al. 2007).

Affordability refers to households’ capability to repay the mortgage loan^6^. Given a certain wealth, households find themselves confronted with limited options of the funding channels. The extent to which a mortgage loan can be easily approached or the flexibility of lending schemes therefore determines households’ affordability, which in turn affects home-occupancy. The characteristics of regulated markets are not the only determinants of mortgage schemes. Nonetheless, this regulatory framework should still be taken into account because of the wide range of mortgage systems. The US may have a more flexible mortgage system than the European countries, resulting from the inherent difference in rules for originating and assembling mortgage loans under the supervision of federal agencies (Coles and Hardt 2000). Though all European lenders are subject to the same EU rules, regional financial intermediaries offer a diverse portfolio of mortgage loans on different markets (Aalbers 2012).

Another aspect of interventions is taxation. Fiscal policy affects affordability by subtly raising or lowering the level of household wealth. Thereby, the tax regime acts as an incentive or disincentive for households to enter the owner-occupied market. A more obvious impact of fiscal policy is demonstrated by the user-cost rate (Poterba 1992). Though many countries have implemented tax deductions, the policies vary largely in their depth and longevity.

The fourth aspect is the intervention of supply side, which strongly affects the construction activities. The long-run house prices would be solely determined by the quantity of housing supply, given a perfect elasticity. This assumption, however, is not realistic, due to the prolonged construction activities. The supply elasticity is expected to be further deteriorated in a country with limited land reserves, not to mention the extra limitation on land development. It has been suggested that supply elasticity is negatively associated with the extent to which regulation is imposed on land provision and housing development (Green et al. 2005). Supply elasticity varies widely within regions, ranging from almost zero in the Dutch market (Vermeulen and Rouwendal 2007a, b) to around thirty in the Dallas region in the USA (Green et al. 2005). Regions in face of a scarcity of land and subject to strict zoning plans—such as in the Netherlands, Japan, Singapore, and Hong Kong—would have a lower supply elasticity.

## Results in the Dutch context and discussion

In this section, we examine the performance of the four types of models (asset-pricing, multi-period utility, stock-flow, and repayment) in the Dutch market. We use a sample between 1980 and 2008 with semi-annual frequency. The global financial crisis of 2008 provided an opportunity to examine those models in a changed scenario, and an extended sample period starting from 1980 to 2014 is also applied^7^. Policies for the Dutch market changed significantly in the post-crisis period, which involve aspects such as the mortgage market^8^, tax deduction polices, and planning deregulation (Priemus and Whitehead 2014).

### Presentations in the Dutch market

We use the simple model form discussed in Sect. 2 to model the house prices in the Dutch market.^9^

To implement the *asset*-*pricing model* in the Dutch market, the asset-pricing form in Eq. (2) is considered in logarithmic form:

3 $$\log (P_{t} ) = \log (R_{t} ) - { \log }({\text{UC}}_{t} ),$$

where $P_{t}$ is the real price, $R_{t}$ is the real rent, and ${\text{UC}}_{t}$ is the user cost. This form^10^ can largely represent the principle of asset-pricing in the house price model.

According to the *stock*-*flow model*, we use a reduced linear form

4 $$\log (P_{t} ) = \beta_{0} + \beta_{1} \log ({\text{HS}}_{t} ) + \beta_{2} \log ({\text{UC}}_{t} ) + \beta_{3} \log (Y_{t} ) + \beta_{4} \log ({\text{CC}}_{t} ) + \beta_{5} \log ({\text{HH}}_{t} ),$$

where $P_{t}$ is the real price, ${\text{HS}}_{t}$ is the housing stock, ${\text{UC}}_{t}$ represents the user-cost rate, $Y_{t}$ is the real income, ${\text{CC}}_{t}$ denotes the construction cost, and ${\text{HH}}_{t}$ is the number of households. This form assumes log-linear relations between rents, incomes, demographic factors, and housing stocks in the property market, as well as between rent and user costs in the asset market.

To evaluate the *multi*-*period utility model* empirically, we apply several approximations for the Dutch market. Each homeowner is assumed to occupy an identical single-family dwelling, following Olsen (1987). This allows us to convert the housing expenditure into the house price without changing the rest of the equation. The minimum spending on housing is proxied by the average rents, while minimum spending on other consumption goods is proxied by a constant fraction of the income level.^11^ The approximation leads to a formulation of demand-oriented house prices

5 $$P_{t} = \gamma_{0} + \gamma_{1} Y_{t} + \gamma_{2} R_{t} ,$$

where $P_{t}$ is the real house price, $Y_{t}$ is the real income, and $R_{t}$ is the real rent.

Following Madsen (2012), the *repayment model* suggests the house prices as

6 $$\log (P_{t} ) = \alpha_{1} \log (\varphi_{t} ) + \alpha_{2} \log (Y_{t} ) + \alpha_{3} \log ({\text{HS}}_{t} ) + \alpha_{4} \log (i_{t}^{a} ),$$

where $P_{t}$ is the house price, $\varphi_{t}$ is the maximum borrowing limit, $Y_{t}$ is the income, ${\text{HS}}_{t}$ is the housing stock^12^, and $i_{t}^{a}$ is the adapted mortgage rates. Equation 6 shows that house prices derived from reversing the repayment model are associated with the financial restrictions, housing stock, household income, and the adapted mortgage rates. Financial institutions usually choose a conventional time-invariant ratio as the maximum borrowing limit. In the Netherlands, the ratio is around 25 %, meaning that $\varphi_{t}$ is conventionally regarded as a constant.^13^

We acknowledge that the specification of Eqs. (3–6) can cause potential problems, such as the misspecification of the function form as nonlinear function is not considered, and the issues of omitted variables. These, among others, can be other possible sources of unsatisfactory modeling results.

The main estimation results were derived from an error-correction process as proposed by Engle and Granger (1987). This method combines the long-term effect, which is suggested by economic principle, and the short-term effect that changes in the course of time. Equations 7 and 8 present the modeling process:

7 $$y_{t} = c_{0} + \mathop \sum \limits_{j = 1}^{s} \varphi_{j} x_{jt}.$$

8 $$\Delta y_{t} = \mathop \sum \limits_{i = 1}^{h} \alpha_{i} \Delta y_{t - i} + \mathop \sum \limits_{j = 1}^{k} \mathop \sum \limits_{i = 0}^{m} \beta_{ji} \Delta x_{jt - i} + \gamma *ec_{t - 1} ,$$

Equation 7 is used to generate the long-run effect, depending on the expression formulated with respect to the four models. The discrepancy measured between the actual value and the long-run value indicates the degree of disequilibrium, which is subsequently incorporated into the error-correction model in Eq. (8), representing the degree of disequilibrium in the error-correction term (${\text{ec}}_{t - 1}$). This method relies on an adjusting process underlying the long-run attracting factor and also on the short-term deviations.

Accounting for the post-crisis period, a dummy indicator, *I*(*t* > 2008s1), is introduced in the sample for 1980–2014. A value of one is assigned to the period starting from the second half of 2008. Only the shift in the constant term is considered due to the restriction on the sample size.

The data used in this study are from different Dutch organizations. Information on house prices was provided by the Netherlands’ Association of Real Estate Agents (Nederlandse Vereniging van Makelaars, NVM). Average rents were obtained from the Ministry of the Interior and Kingdom Relations (Ministerie van Binnenlandse Zaken en Koninkrijksrelaties, BZK), which were further deflated by the price level in 2006 to get the real average rents. The number of households and units in the housing stock, construction costs, and inflation were all generated from data provided by Statistics Netherlands (Centraal Bureau voor de Statistiek, CBS). Household incomes were obtained from the Netherlands Bureau for Economic Policy Analysis (Centraal Planbureau, CPB). Information on mortgage interest rates came from the National Bank (De Nederlandsche Bank, DNB).

Considering the tax deduction for mortgage interest payments, an effective mortgage rate^14^ is calculated by following Van den Noord (2005) such that

9 $$i^{e} = i^{m} - 0.6(i^{m} - 0.0125),$$

where $i^{e}$ represents the effective mortgage rates after deducting the mortgage interest by using a top marginal tax rate, $i^{m}$ is the mortgage interest rate obtained from CPB. In the repayment model, the adapted mortgage interest rate $i_{t}^{a}$ is used by adding the constant loan-to-value ratio^15^ 1.16 to the effective mortgage rate.

The user-cost rate is calculated as the sum of the effective mortgage rate, a property tax of 0.3 %^16^, the depreciation and maintenance rate of 4 %, and the capital gains on housing obtained through averaging the price changes over the past 5 years.^17^

### Results from the conventional models

Table 1 shows the variables used for each model. These fundamentals were applied to reach equilibria, which were subsequently used to formulate an error-correction presentation. Integration order and cointegration as prerequisites were approved by the augmented Dickey–Fuller tests.^18^

**Table 1:** Selected model forms

Variables and models	Rents (*R*)	User costs (UC)	Income (*Y*)	Household numbers (HH)	Housing stock (HS)	Construction costs (CC)	Adapted mortgage rate (*i* ^a^)
Asset-pricing	√	√					
Stock-flow		√	√	√	√	√	
Multi-period utility	√		√				
Repayment			√		√		√

*Note*: All variables appear in real terms, deflated by the base year 2006

Table 2 and 3 summarize the error-correction results. The error-correction form of each model has been achieved for the same criteria by applying the general-to-specific procedure, prior to which the Schwarz information criterion (SIC) was used to select the lags.

**Table 2:** Long-run associations of the four models

	1980–2008				1980–2014			
	Asset-pricing	Stock-flow	Multi-period utility	Repayment	Asset-pricing	Stock-flow	Multi-period utility	Repayment
*c*	5.1***	−8.7	−895,450***	−59***	5.2***	−5.7	−916,404***	−65***
log(*R*)	1.2***	–	–	–	1.2***	–	–	–
log(UC)	−0.37***	−0.17***	–	–	−0.37***	−0.21***	–	–
log(HS)	–	5.8**	–	1.6***	–	0.13	–	1.6***
log(CC)	–	0.9***	–	–	–	1***	–	–
log(*Y*)	–	1.5**	–	4.5***	–	1.6***	–	5.0***
log(HH)	–	−5.7***	–	–	–	−0.3	–	–
*R*	–	–	522***	–	–	–	500***	–
*Y*	–	–	30***	–	–	–	31***	–
log(*i* ^a^)	–	–	–	−0.09	–	–	–	−0.008
*I*(*t* > 2008s1)	–	–	–	–	0.2***	−0.12**	−43,085***	0.27***

*Note*: Asterisks denote different significance levels, with ***, **, * indicating a significance level of 1, 5, and 10 %, respectively

**Table 3:** Results from error-correction forms

	1980–2008				1980–2014			
	Asset-pricing	Stock-flow	Multi-period utility	Repayment	Asset-pricing	Stock-flow	Multi-period utility	Repayment
erc	−0.15**	−0.29***	−0.02	−0.038*	−0.14**	−0.19***	−0.03	−0.04**
∆log(R)	0.86**	–	–	–	0.9**	–	–	–
∆log(UC)	−0.15***	−0.14***	–	–	−0.14***	−0.13***	–	–
∆log(HS)	–	1.2	–	–	–	−0.44	–	–
∆log(CC)	–	1.3***	–	–	–	0.93**	–	–
∆log(Y)	–	0.85*	–	0.90***	–	0.95**	–	0.94***
∆log(HH)	–	−2.4	–	–	–	−0.43	–	–
∆HP(−1)	–	–	0.77***	–	–	–	0.61***	–
∆*R*	–	–	648***	–	–	–	677***	–
∆*R*(−1)	–	–	−458***	–	–	–	−327*	–
∆log(HP(−1))	–	–	–	0.67***	–	–	–	0.61***
Δlog(Y(−1))	–	–	–	– 0.75**	–	–	–	−0.53*
Δlog(*i* ^a^(−1))	–	–	–	−0.21***	–	–	–	−0.26***
*I*(*t* > 2008s1)	–	–	–	–	−0.014	−0.014	−2328*	−0.01*

*Note*: Asterisks denote different significance levels, with ***, **, * indicating a significance level of 1, 5, and 10 %, respectively

All the coefficients present the expected signs, except for those of the housing stock and the demographic factor. Their unexpected signs may be linked to the explanation of changes in the number of households. In fact, that number may not constitute the effective housing demand, as newly formed households usually belong to the younger age cohort, whose entry into owner-occupancy is restricted. The opposite direction of the influence of the housing stock may be caused by neglecting the influx of housing in the Dutch market. The asset-pricing approach seems inappropriate, as no evidence has been found of the restricted association between rents and user cost indicated in Eq. (3), based on the Wald test. The generated income-to-price elasticity varies widely, particularly in the repayment model. An income elasticity of 4.5 is much higher than that in studies by the OECD (2004) and Kranendonk and Verbruggen (2008), which reported 1.94 and 1.53, respectively. This uncommonly high-income elasticity indicates the repayment model alone is not sufficient to characterize the Dutch market. Interestingly, the mortgage rates indicate significant influence only in the short run, contradicting findings in the literature on the Dutch market.^19^

Table 4 summarizes the adjusted *R* squared ($\overline{R}^{2}$), the standard error of regression estimation (SE), and the Akaike information criterion (AIC). The sample including the post-crisis period shows error-correction modeling results comparable to that of the before the crisis: a slight decrease of modeling fit appears in the stock-flow model and the multi-period utility model. The four models presented a relatively high $\overline{R}^{2}$ in the long run, falling into the range with an upper limit of 0.97 and a lower limit of 0.75. A variation occurs in the results of four error-correction forms. For instance, during 1980–2008, the $\overline{R}^{2}$ of the asset-pricing model and the stock-flow model is less than 0.40, compared to that for the multi-period utility model (0.69) and for the repayment model (0.72). The relatively higher fit of the repayment modeling result is in part associated with that the Netherlands has an active mortgage market with a high loan-to-value ratio. This result is also consistent with the regression error and AIC, reporting the lowest value for the repayment model.

**Table 4:** Indicators for the modeling results

	1980–2008				1980–2014			
	Long-run $\overline{\text{R}}^{2}$	ECM $\overline{\text{R}}^{2}$	SE	AIC	Long-run $\overline{\text{R}}^{2}$	ECM $\overline{\text{R}}^{2}$	SE	AIC
Asset-pricing	0.94	0.36	0.03	−4.0	0.94	0.36	0.03	−4.1
Stock-flow	0.97	0.37	0.03	−4.1	0.96	0.30	0.03	−3.9
Multi-period utility	0.79	0.69	3078	18.9	0.79	0.55	4052	19.5
Repayment	0.75	0.72	0.02	−4.9	0.78	0.69	0.02	−4.8

The interval of the $\overline{R}^{2}$ becomes slightly larger when the range of the sample is extended to 2014. This result somehow contradicts our expectation of a remarkably lowered modeling fit in the larger sample period, which should have been caused by the policy changes and their influences on all the parties in the market. However, the slight discrepancy may be attributed to the manner in which shifts enter the model. The changes can take various forms, including elasticity of income. However, this aspect is ignored in the empirical study, primarily because of the small sample size for the post-crisis period.^20^ Other evidence can be found in the unexpected sign of the dummy variable in the asset-pricing model, but also in the relatively stable parameters of these two samples.

The unsatisfactory results validate our hypothesis that the asset-pricing model and stock-flow model do not fit well in the Dutch market, given the competitive market assumption embedded in these two types. Though the multi-period utility type has a good fit, the model itself is problematic as the error-correcting term is not significant at the least acceptable level. By implication, disequilibrium would accumulate over time without any adjustment; this model thus cannot explain the price dynamics. The repayment model appears to be the best of the four, given the strong reliance of the Dutch market on financial affordability.

The four models illustrate different rates of adjustment, mainly in two categories. The asset-pricing model and stock-flow model fall into the larger interval. More moderate adjustments of disequilibrium emerge in the other two models at a comparable level: around 2 % of the disequilibrium is corrected by the multi-period utility model, around 4 % by the repayment model.

**Fig. 1:**
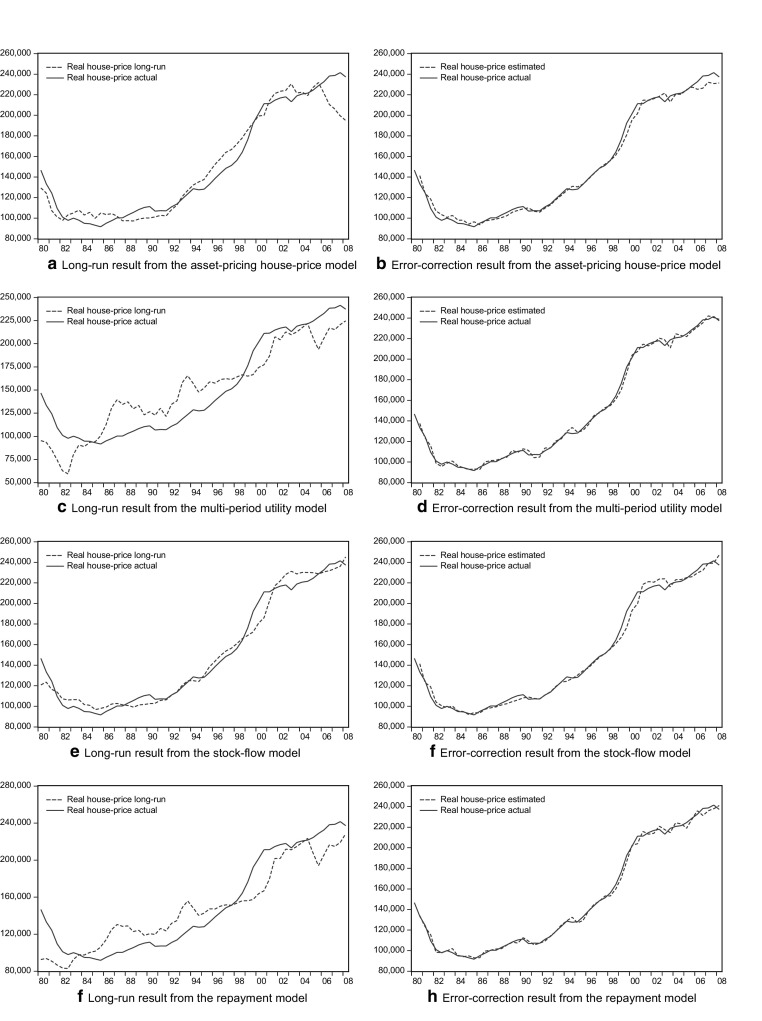
Modeling results through 1980-2008

**Fig. 2:**
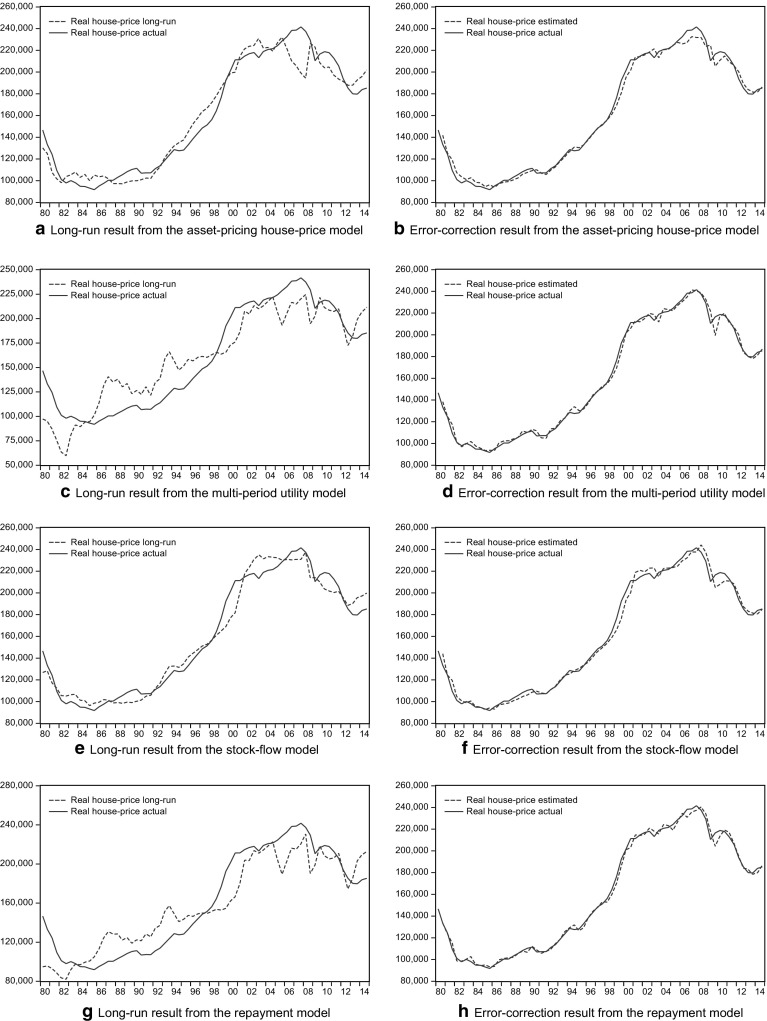
Modeling results through 1980-2014

Figures 1 and 2 present house prices in the long and short run according to each type of model in the sample periods 1980–2008 and 1980–2014. The long-run trend in the stock-flow model seems to oscillate closely around the actual price movement within a smaller bound, suggesting better fit in the long term. The long-run relationships of the four models also provide a different insight into whether house prices are overvalued in a given period and how the trends pass on to the next period. Similarities emerge among the models, especially between the asset-pricing and stock-flow type and between the multi-period utility and the repayment type. The models differ in their over/under-valuation of house prices. Specifically, the multi-period utility model and the repayment model suggest a larger variation in the magnitude of the over/under-valued part of house prices. Further adjustment is therefore more likely to appear when the over-valued house price reaches a certain amount. For example, house prices reached their peak in the second half of 2007^21^ before the crisis hit the market, but they were not as much overvalued as house prices had been in 2006.

**Fig. 3:**
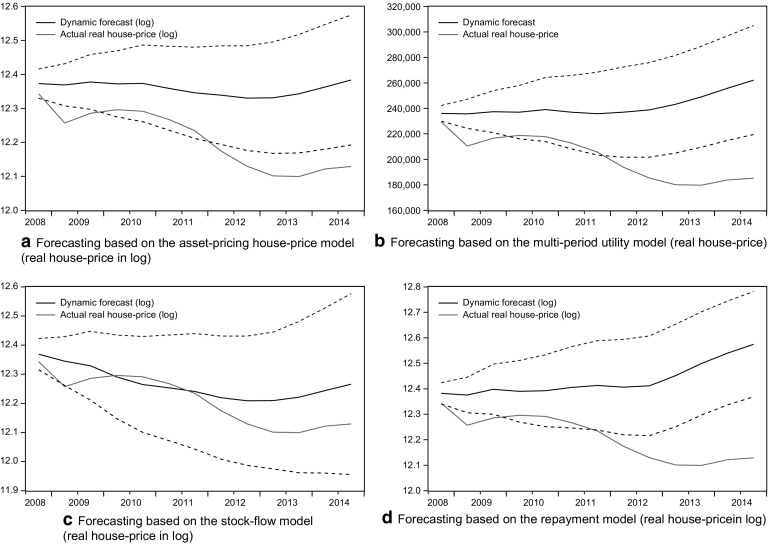
Forecasting based on the 1980–2008 estimation. All forecasted prices are in logarithmic form, except for the multi-utility model, as the presentation leads directly to the absolute price level. The *dashed lines* suggest the 95 % confidence interval in each sub-panel

Figure 3 depicts the forecasted house prices based on the main estimation (1980–2008). As expected, the discrepancy between the forecasted prices and real prices is considerable, especially for the asset-pricing, multi-period utility, and repayment types. And the forecasted confidence interval does not cover most parts of the real price dynamics.

The modeling results provide evidence that the asset-pricing type and stock-flow type do not fit the Dutch market, while the multi-period utility type and repayment type seem to perform better. However, there are problems with the latter two models as well. That is because these models cover the market characteristics only partially, implying that it is necessary to integrate market characteristics in a regulated environment.

### Further discussion on the conventional models

The unsatisfactory empirical results give us the motivation to search answers from the conventional models. Though the asset-pricing model has a sounded economic ground and provides a straightforward intuition, it suffers several disadvantages which could lower the power of this type of model. The asset-pricing model of house prices assumes a stationary rent-to-price ratio as time moves toward infinity, which indicates that a smaller house price appreciation occurs immediately after a sharp increase in house price, vice versa. The economic reason of this ex ante stationary rent-to-price ratio, however, is lacking and the strong association was questioned by Campell and Shiller (1988a, b). Another demerit is that the asset-pricing model excludes the impact from the credit market and assumes a perfect credit market.

The stock-flow house price model provides a dynamic view to connect different states of equilibria, which in turn can also become a demerit. This model presumes a quick market clearing, meaning that the changes in demand of every period would be absorbed quickly through the stock in the same period.^22^ The model therefore excludes the scenario in which there is demand surplus. This model also has the disadvantage shared by the asset-pricing model. Efforts have been made to partially modify the stock-flow model.

The multi-period utility model focuses on households’ behavior and provides a reasonable method to incorporate decision-making process. This demand-driven model may not be able to explain equilibrium in the long run, but can be appropriate for markets such as the Netherlands where the supply side is strongly regulated. However, the generalization of the result is difficult to achieve as it depends on the choice of the utility realization.

The repayment model bridges lending rules and households who possess the demand in ownership, which in turn affects lending stringency. Still, it has some drawbacks. Treating the lending rules as time-invariant strictly excludes the possibility of the time-variant structure changes. Consequently, any dramatic change in the macro condition is ruled out. This model also tends to emphasize the dominant role played by lenders and assumes that borrowers would take up the maximum amount allowed. Thus, households have less market power in this model. A repayment model reflects the condition of housing finance. Thus, this type of model fits markets that rely strongly on banking finance, such as the Dutch market. In a 2004 study, Boelhouwer and others proposed an affordability model (or repayment model) for the owner-occupied market in the Netherlands. They suggested that an interest-to-income ratio, rather than price-to-income, could represent this market adequately.^23^ This particular repayment model, however, captures mainly the regulatory characteristic of financial affordability.

Table 5 summarizes the regulatory aspects that the four classic models have addressed. Each model has partially covered the interventions, instead of all the four aspects; this provides the possible source of the unsatisfactory performances when the four models are directly applied to a regulated market.

**Table 5:** Regulatory characteristics addressed in the four models

Regulatory characteristics and the four models	Tenure	Affordability	Tax regime	Supply activities
Asset-pricing	√		√	
Stock-flow	√		√	√
Multi-period utility		√	√	
Repayment		√	√	

These four models emphasize different aspects. By focusing on competitive markets, the models call attention to the potential problems of a regulated environment. The stock-flow model has taken account of the supply activities. However, by forming the price in the asset market, and thereby sharing a property of the asset-pricing model, the stock-flow model is less suitable for a regulated environment. Both the multi-period utility model and the repayment model highlight some market features from the demand side; they are thus expected to partially capture the regulatory characteristics.

### Channels through interventions to house prices

The influence of the interventions—tenure, financial affordability, tax regime, and supply—on price-formulating process is realized in different ways. The structure of the rental market determines the accessibility of renting, which is viewed as a substitute for home ownership, indirectly influencing house prices (King 1980). Renting is sometimes regarded as the only choice for those who fail to satisfy the financial requirement of becoming an owner, among other restrictions. For this group of households, the substituting function of owning is temporarily weakened. The situation is, however, different in markets with a subsidized rent system through either restricting the rent ceiling and controlling the increase of rents or directly providing social housing, such as in most European countries, or by combing both, such as in the Netherlands. Under such conditions, renting as a substitute for owning seems more beneficial, potentially imposing a downward pressure on house prices, given sufficient subsidized rental units. This downward pressure on house prices is, however, alleviated as subsidized renting could possibly increase the households’ saving, helping build up the capital accumulation required to enter the ownership.

**Fig. 4:**
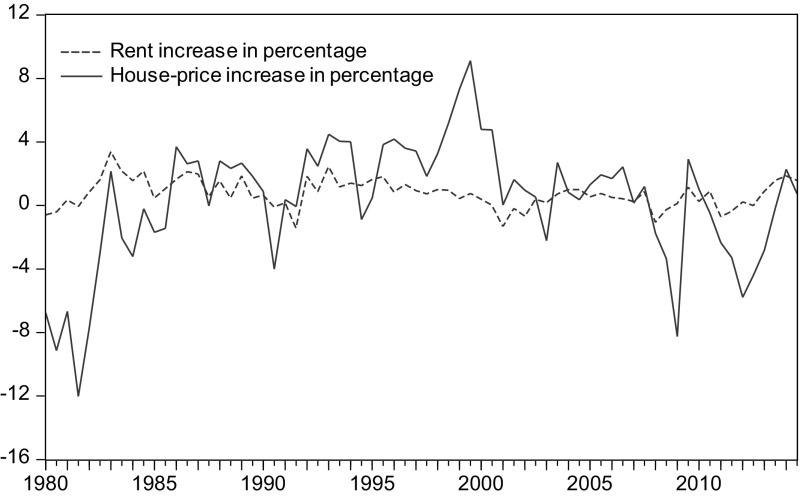
Increases in house prices and rents in the Netherlands

The Netherlands has a highly regulated rental market, with over 75 % of the rental sector in social renting in 2012. Figure 4 shows the changes of house prices and rents for the past 30 years, and it is obvious that rental market is strongly regulated as the increase of rents stays within a narrow range. Despite such a high proportion of social renting dwellings, the position of renting as an alternative to owning has not been strengthened. One reason is the severely compressed private rental market that results from the considerable provision of social housing and the policy guidance leaning toward owner-occupancy (Boelhouwer and Van Der Heijden 1992; Haffner 2011). Households now find themselves in a squeeze: among the tenures—social renting, free-market renting, and owning—which are supposed to become more accessible than the way it turns out, considering the long waiting list in the social rental sector, the considerable capital requirement to enter home ownership, and the high rents in the free rental sector. The dilemma becomes most challenging for the young people who have insufficient capital to buy a dwelling and suffer the difficulty in approaching social housing. Intervention in the rental sector does not make substitution easier, but rather tends to curb the tenure options (leave fewer choices) for households and could possibly push up the house prices to a higher level than these should be in an unregulated market.

Affordability determines whether households who are indifferent between owning and renting or who prefer owning over renting are capable of becoming homeowners. As defined here, affordability falls into the realm of financial feasibility, which is determined mainly by a household’s income level and the lending criteria imposed by the financial institutions. The variation among mortgage schemes is highlighted as the diversity provides additional flexibility for households, offering options that lower the entry threshold and thereby ease access to ownership. Presumably, a more flexible mortgage system would lead to a prosperous housing market (Aalbers 2008).

The tax policy that allows the taxpayer to deduct mortgage interest payments accelerates the booming housing market in the Netherlands, in accompany with financial liberalization in the 1990s. The tax regime is carried out because of the government’s housing targets, such as promoting home ownership, and stimulating extra housing demand. One way to look at the influence is from user cost in which tax deduction rate appears as a component. The cost of owning is reduced in the presence of tax deduction because the effective mortgage interest is reformulated to a more favorable level. As expected, households are motivated to prone toward the owner-occupied sector, pushing up house prices (Poterba 1984).

**Fig. 5:**
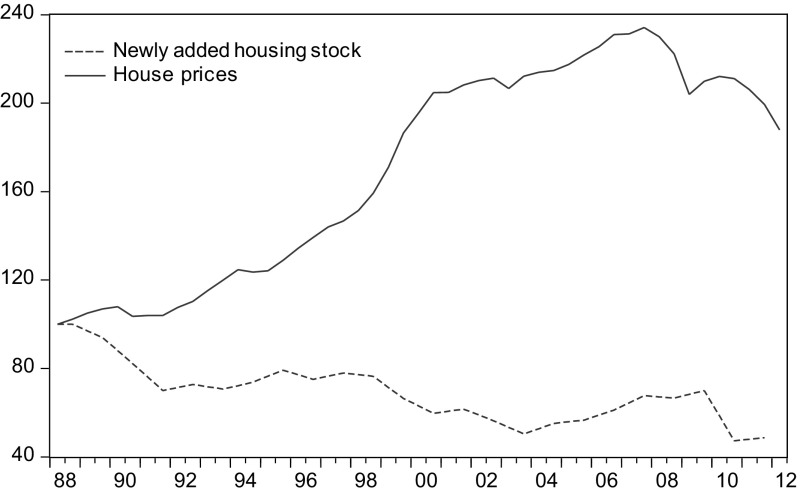
Gap between the newly added housing stock and real house prices in the Netherlands (index base year 1988S1 = 100)

The intervention of the housing supply sector helps accelerate the increase of house prices. If the time frame is sufficiently long, housing supply ultimately determines the price level since supply side controls the speed of production so that the quantity of extra added housing units would be adjusted to absorb the changes in demand. This influence implies that house price volatility is proportionate to the elasticity of the supply. In a similar manner, any regulations that jeopardize its elasticity would induce more fluctuations in house prices (Glaeser et al. 2008). This situation is commonly found in highly regulated markets, where positive demand shocks are absorbed only to a limited degree. In light of the observation from the Dutch housing market, a considerable gap exists between the newly added units in the housing stock and the house prices, suggesting a mismatch of the stock-flow model.^24^ As shown in Fig. 5, despite a volatile development of house prices over the past 30 years, the newly added housing has remained more or less steady. In addition, Vermeulen and Rouwendal (2007a, b) found an almost zero supply elasticity of house prices in the Netherlands.

## Conclusion and future work

The aim of the study is to examine whether a mismatch exists between standard house price models and the markets under interventions and to provide the possible reasons behind, if any. To that end, house price models were divided into four types: asset-pricing models, multi-period utility models, stock-flow models, and repayment models. These types were distinguished on the basis of their theoretical emphasis and underlying assumptions.

The models were compared empirically in the Dutch market. The results verified the mismatch between the conventional models and the regulatory environment. However, the multi-period utility model and repayment model showed a smaller degree of mismatch than that of the asset-pricing and the stock-flow models. Though insights are generated from the Dutch market, it is expected to see similar results of mismatch other regulated markets.

The general mismatch reflects the neglects of the aspects of interventions which, in this study, were decomposed into tenure choice, financial affordability, tax regime, and supply-side activities. These regulatory elements functioned as the driving force of an anticipated mismatch between the regulatory environment and conventional models which typically neglect regulatory features. The regulatory characteristics, however, are not negligible because of the different influences they have on households’ decision-making process, which in turn determines the dynamics of house prices. In other words, the tenure which highlights the option constraints that households confront, the financial affordability which indicates influences exerted by financial intermediaries, the tax regime which suggests policy guidance, and the supply activities which reflect the market flexibility, simultaneously play a role in a regulated housing market.

To better model house prices in the regulated markets, one needs to take account of the regulatory characteristics. The supply-associated regulation, for instance, can be captured by modifying the supply function. Affordability and the tax regime need to be combined in the households’ utility-maximizing problem, which can be represented by the financial constraint confronted by the households. An integrated framework of modeling regulated markets is thus required in future work.

Potential improvement could be made by capturing a wider range of regulated features, notably by elaborating on housing finance, though it is challenging. This study is the first step toward house price modeling in a regulated environment. Its value lies in demonstrating a way to relate competitive markets to regulated ones. However, we acknowledge that different results that are comparable may arise when putting different strategies of modeling to practice, depending on how each step in the analysis is implemented and how accurate the measurement is.

## Footnotes

1 It is challenging to give a precise definition of regulation for the housing market due to the complexity and potential interaction of different regulatory aspects.

2 Our aim is not to propose a division that is absolutely incompatible, but rather a division of house price models that stresses a particular economic aspect. Other ways of distinguishing the models are also possible.

3 Though the choice of utility form would affect the final expression of house prices, given the family of different utility functions, the Stone–Geary utility form is selected with the intention to illustrate the mechanism to explain house prices from this economic perspective. We believe the Stone–Geary utility function is an appropriate representation for the housing preference not only because it has taken the minimum amount of consumption into account, but also it has relatively simple form. We, however, keep an open mind for other possibilities.

4 Although the definition of regulated markets is lacking in literature, we intend to focus on four aspects in this study, namely, tenure, affordability, taxation, and supply.

5 Here tenure refers to alternatives to ownership or substitutions for owner–occupancy.

6 Affordability in our discussion only refers to the easiness to finance mortgage, though it has a different meaning in welfare field.

7 The same presentations are applied in the extended sample, except for the dummies that were introduced to suggest the post-crisis period.

8 For example, a gradually lowered maximum loan-to-value ratio is targeted at 100 % for 2018 and was set at 104 % in 2014 by law.

9 We provide the economic principle of each model type instead of the derivation of each empirical equation in this study. The details of derivation can be found in the corresponding literature mentioned in the paper.

10 Kim (2007) discussed how the rent-to-price ratio is determined when the loan-to-value ratio is binding. The Netherlands had a portfolio loan-to-value (calculated as the ratio of all the mortgage debt and the value of dwellings of mortgage borrowers) of 72 % in 2013 (according to the Dutch Banking Association), suggesting an unbinding loan-to-value constraint, or in other words, the ceiling of loan-to-value is not reached.

11 We acknowledge that the figures for minimum spending on these two categories are rough approximations.

12 Housing stock enters Eq. (6) as a result of an aggregation of the average consumption of housing size for the total households.

13 However, this ratio reflects an aggregate trend derived from financial regulations, monetary policies, government attitudes, and economic cycle, suggesting a time-dependent $\varphi_{t}$.

14 We follow the calculation by Van den Noord (2005) to consider the impact of imputed rent, which is taxed at 1.25 % of the housing value with a top marginal rate of 60 %. Though the tax regime has experienced some variation, we stick to this proxy.

15 Due to data limitations, we use the average loan-to-value ratio over the period 2005–2012, which was generated by dividing the amount of the mortgage by the transaction price, which we drew from the data of the BZK.

16 The property tax rates vary from 0.1 to 0.9 %, depending on local rates (Van den Noord 2005). We choose the rate of 0.3 % as an average because most municipalities tend to apply tax rates in a lower range.

17 This measurement follows Poterba (1984).

18 We did not report the tests because it is less a concern for this study, but the results of these tests can be approached at request.

19 See, for instance, Kranendonk and Verbruggen (2008) and De Vries and Boelhouwer (2009) for more details.

20 Introducing extra change in one parameter would lead to at least two additional coefficients.

21 IMF (2005) also suggests an overvalued house price in 2007.

22 DiPasquale and Wheaton (1994) have taken into account not only the sluggish adjustment in the housing stock but also the price adjustment caused by changes in demand—an extra adjustment rate was introduced resembling the speed of price changes. Although the gradual adjustment in the house price mechanism seems to contribute only to a limited extent to the equilibrium state, the adjustment does provide a way to incorporate house prices from the previous period as a component of the short-term effects. This can be seen from the lags in house prices that are used to represent part of the market anticipations. It is assumed that a rational individual would utilize all available information to minimize systematic mistakes.

23 Francke et al. (2009) argued that the interest-to-income ratio was nonstationary. This, however, is not necessarily against the repayment model type.

24 Notably the housing stock seems to move in line with demographic change, which suggests a balance between the housing flow and the ‘demographic demand.’ Yet the latter is not necessarily equivalent to the real housing demand. The housing demand differs from the demographic change for reasons such as, to mention a few, the preference change in the tenure choice, which leads to extra demand for owner–occupancy, and the expectation of higher house prices, which stimulates more potential home buyers to take action. Another evidence of the gap between newly added housing and the house prices is the supply elasticity of house prices.
